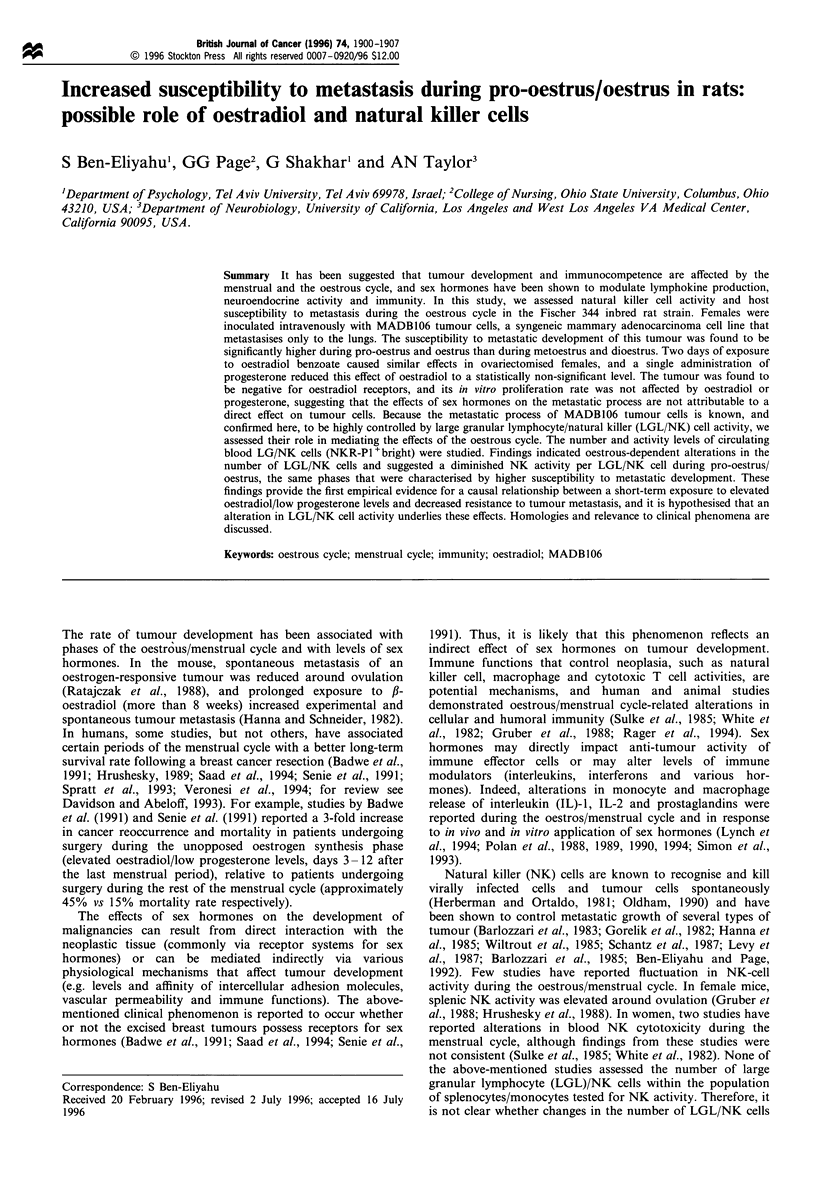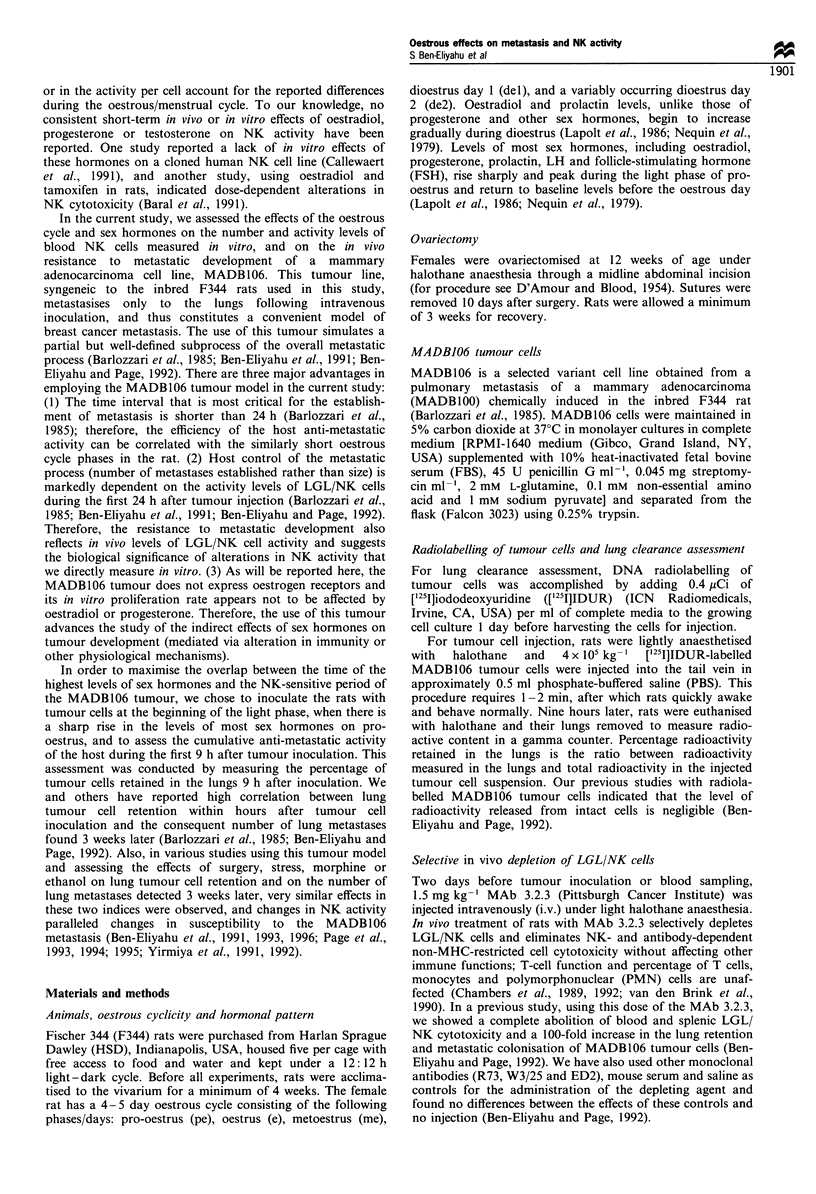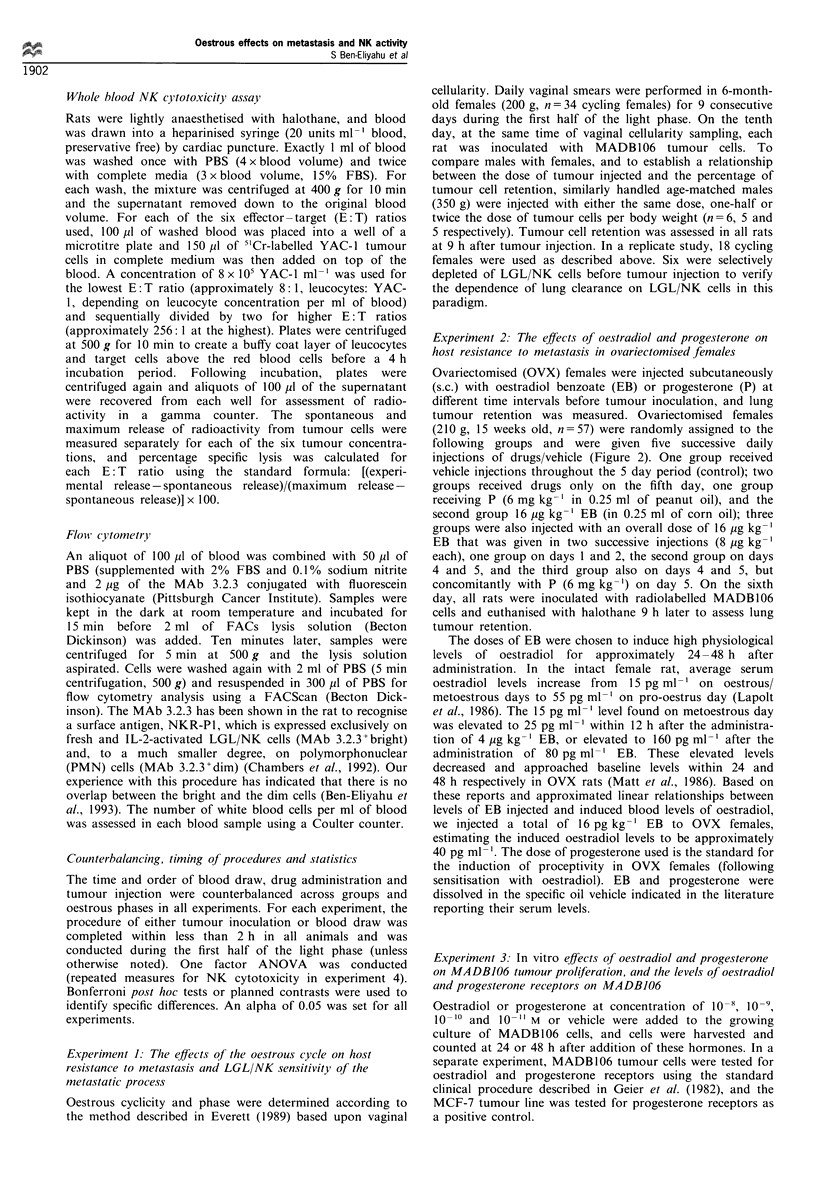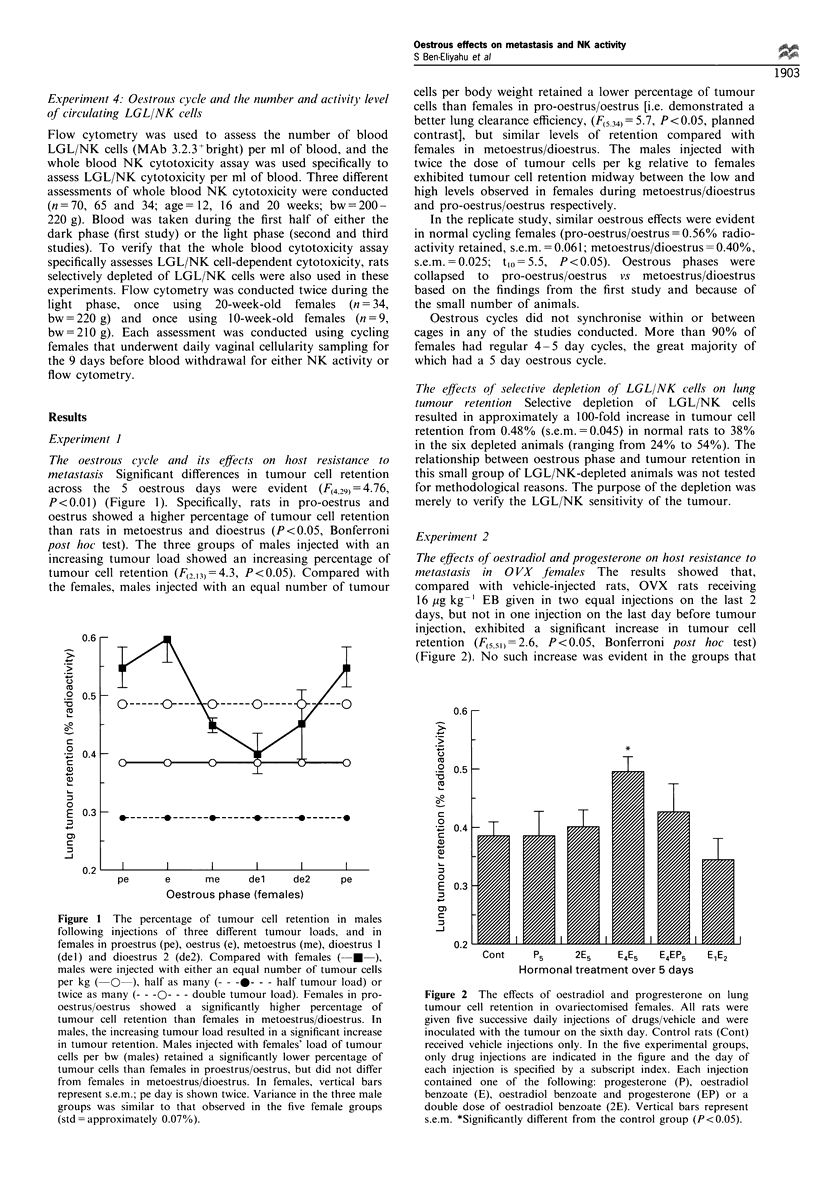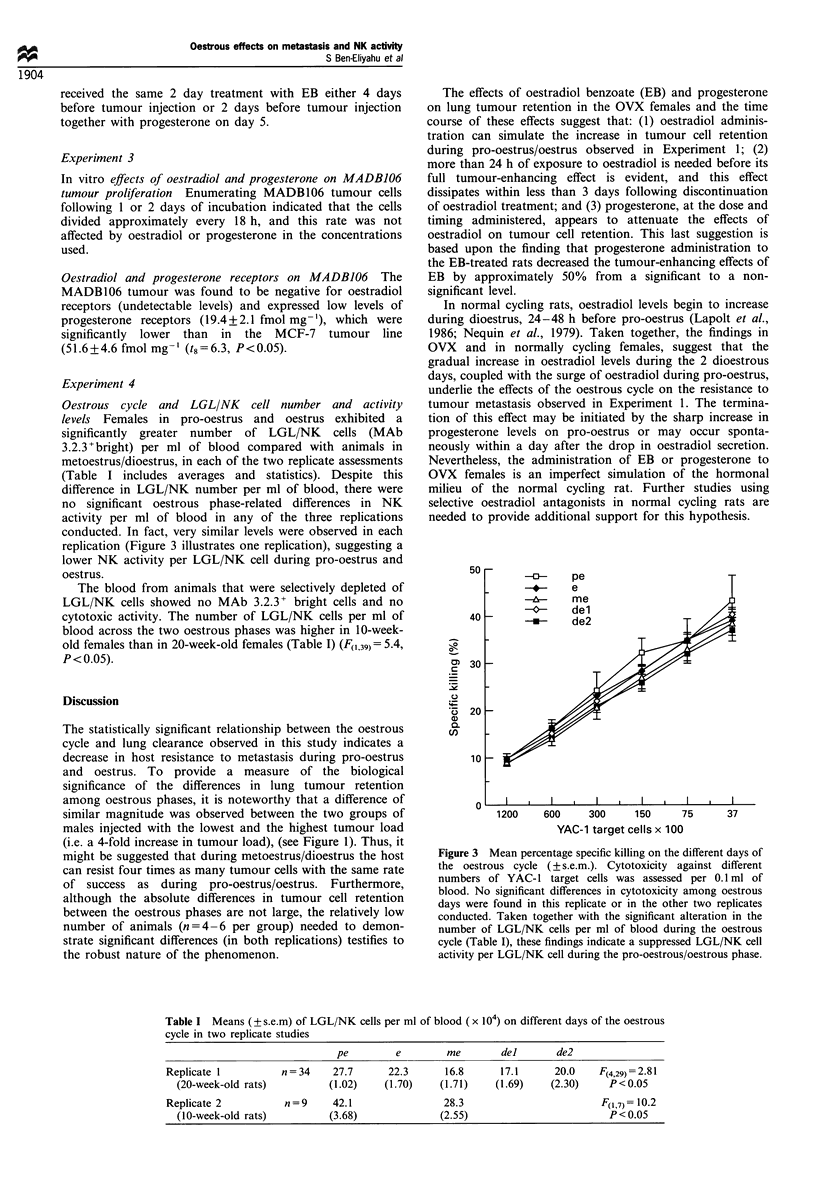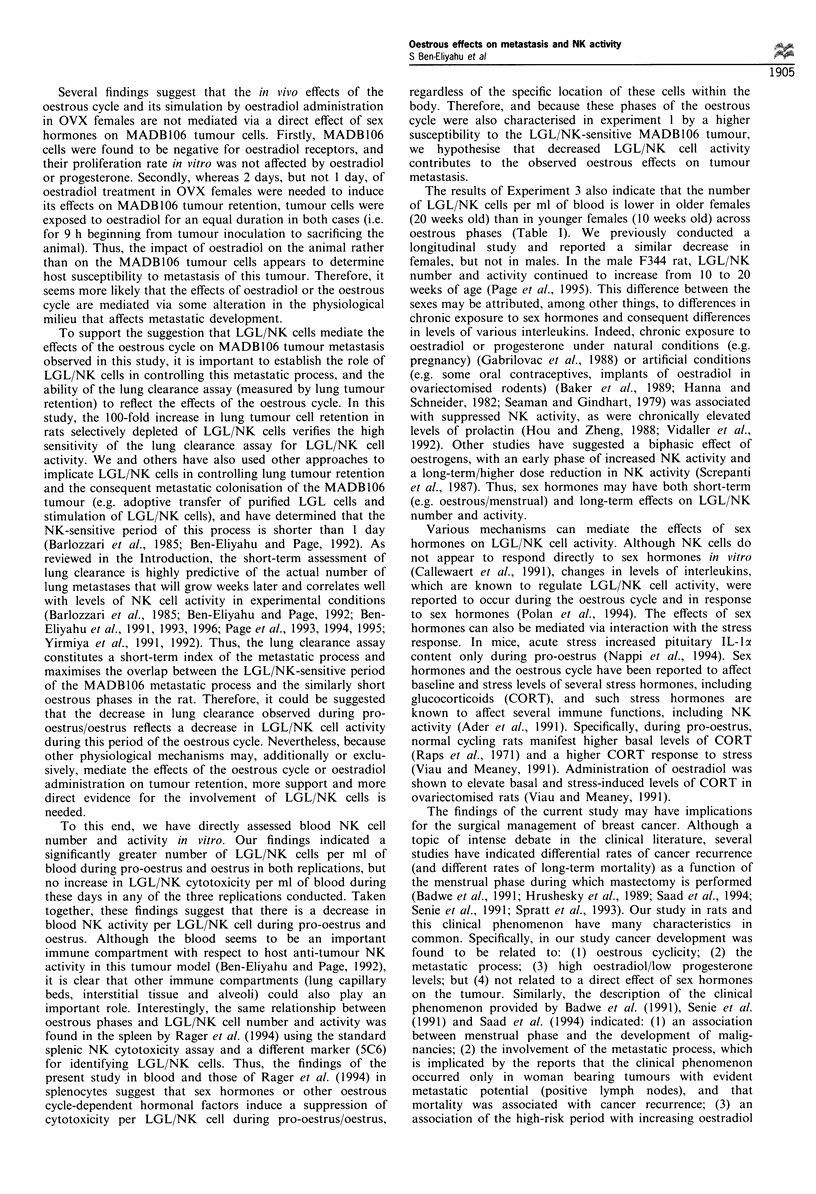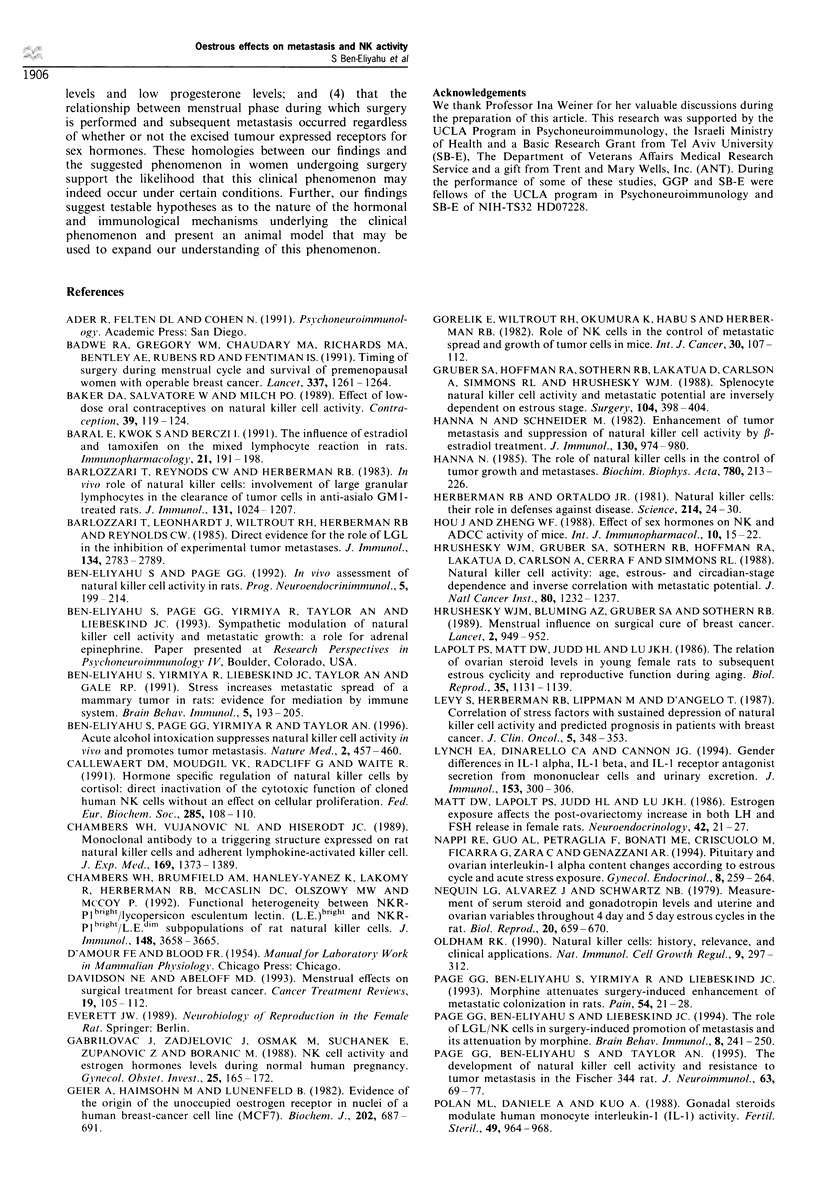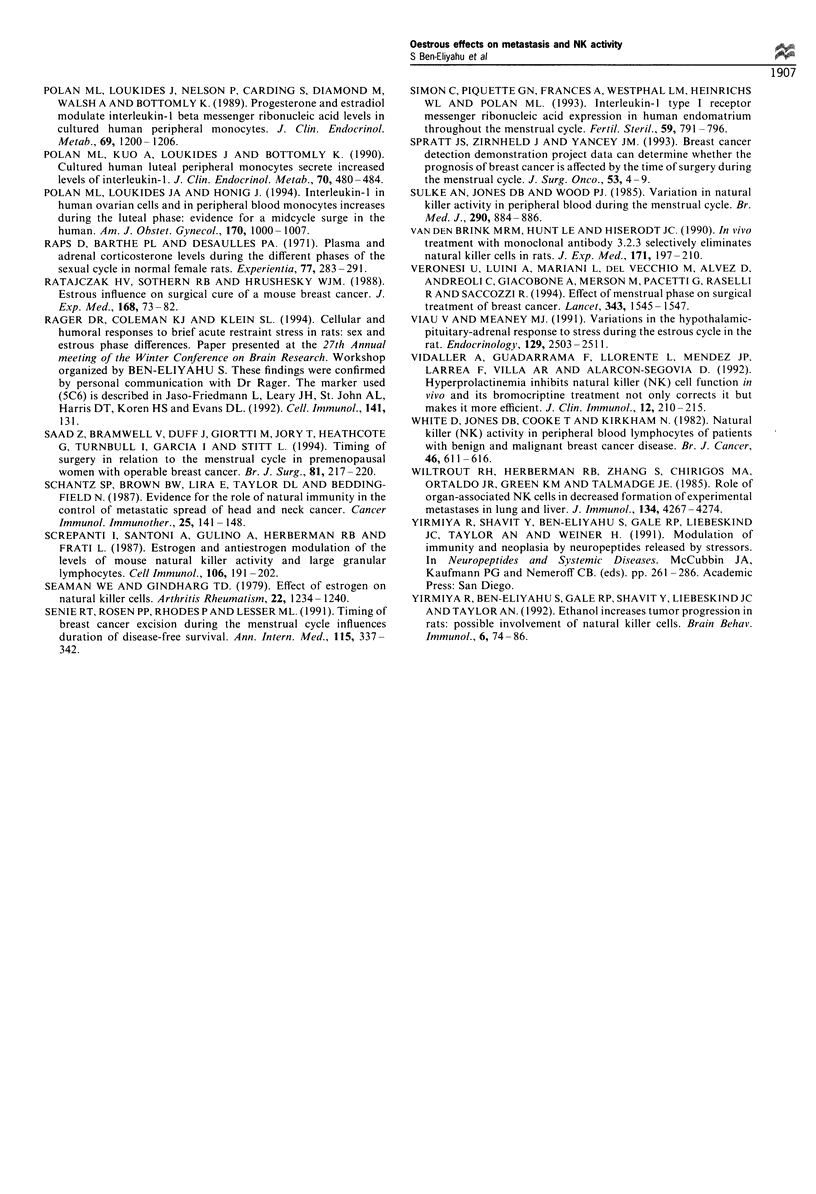# Increased susceptibility to metastasis during pro-oestrus/oestrus in rats: possible role of oestradiol and natural killer cells.

**DOI:** 10.1038/bjc.1996.651

**Published:** 1996-12

**Authors:** S. Ben-Eliyahu, G. G. Page, G. Shakhar, A. N. Taylor

**Affiliations:** Department of Psychology, Tel Aviv University, Israel.

## Abstract

It has been suggested that tumour development and immunocompetence are affected by the menstrual and the oestrous cycle, and sex hormones have been shown to modulate lymphokine production, neuroendocrine activity and immunity. In this study, we assessed natural killer cell activity and host susceptibility to metastasis during the oestrous cycle in the Fischer 344 inbred rat strain. Females were inoculated intravenously with MADB106 tumour cells, a syngeneic mammary adenocarcinoma cell line that metastasises only to the lungs. The susceptibility to metastatic development of this tumour was found to be significantly higher during pro-oestrus and oestrus than during metoestrus and dioestrus. Two days of exposure to oestradiol benzoate caused similar effects in ovariectomised females, and a single administration of progesterone reduced this effect of oestradiol to a statistically non-significant level. The tumour was found to be negative for oestradiol receptors, and its in vitro proliferation rate was not affected by oestradiol or progesterone, suggesting that the effects of sex hormones on the metastatic process are not attributable to a direct effect on tumour cells. Because the metastatic process of MADB106 tumour cells is known, and confirmed here, to be highly controlled by large granular lymphocyte/natural killer (LGL/NK) cell activity, we assessed their role in mediating the effects of the oestrous cycle. The number and activity levels of circulating blood LG/NK cells (NKR-PI+ bright) were studied. Findings indicated oestrous-dependent alterations in the number of LGL/NK cells and suggested a diminished NK activity per LGL/NK cell during pro-oestrus/ oestrus, the same phases that were characterised by higher susceptibility to metastatic development. These findings provide the first empirical evidence for a causal relationship between a short-term exposure to elevated oestradiol/low progesterone levels and decreased resistance to tumour metastasis, and it is hypothesised that an alteration in LGL/NK cell activity underlies these effects. Homologies and relevance to clinical phenomena are discussed.


British Journal of Cancer (1996) 74, 1900-1907
© 1996 Stockton Press All rights reserved 0007-0920/96 $12.00

### Increased susceptibility to metastasis during pro-oestrus/oestrus in rats: possible role of oestradiol and natural killer cells

S Ben-Eliyahu[1], GG Page[2], G Shakhar[1] and AN Taylor[3]

[1]*Department of Psychology, Tel Aviv University, Tel Aviv 69978, Israel;* [2]*College of Nursing, Ohio State University, Columbus, Ohio 43210, USA;* [3]*Department of Neurobiology, University of California, Los Angeles and West Los Angeles VA Medical Center, California 90095, USA.*

**Summary** It has been suggested that tumour development and immunocompetence are affected by the menstrual and the oestrous cycle, and sex hormones have been shown to modulate lymphokine production, neuroendocrine activity and immunity. In this study, we assessed natural killer cell activity and host susceptibility to metastasis during the oestrous cycle in the Fischer 344 inbred rat strain. Females were inoculated intravenously with MADB106 tumour cells, a syngeneic mammary adenocarcinoma cell line that metastasises only to the lungs. The susceptibility to metastatic development of this tumour was found to be significantly higher during pro-oestrus and oestrus than during metoestrus and dioestrus. Two days of exposure to oestradiol benzoate caused similar effects in ovariectomised females, and a single administration of progesterone reduced this effect of oestradiol to a statistically non-significant level. The tumour was found to be negative for oestradiol receptors, and its *in vitro* proliferation rate was not affected by oestradiol or progesterone, suggesting that the effects of sex hormones on the metastatic process are not attributable to a direct effect on tumour cells. Because the metastatic process of MADB106 tumour cells is known, and confirmed here, to be highly controlled by large granular lymphocyte/natural killer (LGL/NK) cell activity, we assessed their role in mediating the effects of the oestrous cycle. The number and activity levels of circulating blood LG/NK cells (NKR-P1$^{+}$bright) were studied. Findings indicated oestrous-dependent alterations in the number of LGL/NK cells and suggested a diminished NK activity per LGL/NK cell during pro-oestrus/oestrus, the same phases that were characterised by higher susceptibility to metastatic development. These findings provide the first empirical evidence for a causal relationship between a short-term exposure to elevated oestradiol/low progesterone levels and decreased resistance to tumour metastasis, and it is hypothesised that an alteration in LGL/NK cell activity underlies these effects. Homologies and relevance to clinical phenomena are discussed.

**Keywords:** oestrous cycle; menstrual cycle; immunity; oestradiol; MADB106

The rate of tumour development has been associated with phases of the oestrous/menstrual cycle and with levels of sex hormones. In the mouse, spontaneous metastasis of an oestrogen-responsive tumour was reduced around ovulation (Ratajczak *et al.*, 1988), and prolonged exposure to $\beta$-oestradiol (more than 8 weeks) increased experimental and spontaneous tumour metastasis (Hanna and Schneider, 1982). In humans, some studies, but not others, have associated certain periods of the menstrual cycle with a better long-term survival rate following a breast cancer resection (Badwe *et al.*, 1991; Hrushesky, 1989; Saad *et al.*, 1994; Senie *et al.*, 1991; Spratt *et al.*, 1993; Veronesi *et al.*, 1994; for review see Davidson and Abeloff, 1993). For example, studies by Badwe *et al.* (1991) and Senie *et al.* (1991) reported a 3-fold increase in cancer reoccurrence and mortality in patients undergoing surgery during the unopposed oestrogen synthesis phase (elevated oestradiol/low progesterone levels, days 3–12 after the last menstrual period), relative to patients undergoing surgery during the rest of the menstrual cycle (approximately 45% *vs* 15% mortality rate respectively).

The effects of sex hormones on the development of malignancies can result from direct interaction with the neoplastic tissue (commonly via receptor systems for sex hormones) or can be mediated indirectly via various physiological mechanisms that affect tumour development (e.g. levels and affinity of intercellular adhesion molecules, vascular permeability and immune functions). The above-mentioned clinical phenomenon is reported to occur whether or not the excised breast tumours possess receptors for sex hormones (Badwe *et al.*, 1991; Saad *et al.*, 1994; Senie *et al.*, 1991). Thus, it is likely that this phenomenon reflects an indirect effect of sex hormones on tumour development. Immune functions that control neoplasia, such as natural killer cell, macrophage and cytotoxic T cell activities, are potential mechanisms, and human and animal studies demonstrated oestrous/menstrual cycle-related alterations in cellular and humoral immunity (Sulke *et al.*, 1985; White *et al.*, 1982; Gruber *et al.*, 1988; Rager *et al.*, 1994). Sex hormones may directly impact anti-tumour activity of immune effector cells or may alter levels of immune modulators (interleukins, interferons and various hormones). Indeed, alterations in monocyte and macrophage release of interleukin (IL)-1, IL-2 and prostaglandins were reported during the oestros/menstrual cycle and in response to *in vivo* and *in vitro* application of sex hormones (Lynch *et al.*, 1994; Polan *et al.*, 1988, 1989, 1990, 1994; Simon *et al.*, 1993).

Natural killer (NK) cells are known to recognise and kill virally infected cells and tumour cells spontaneously (Herberman and Ortaldo, 1981; Oldham, 1990) and have been shown to control metastatic growth of several types of tumour (Barlozzari *et al.*, 1983; Gorelik *et al.*, 1982; Hanna *et al.*, 1985; Wiltrout *et al.*, 1985; Schantz *et al.*, 1987; Levy *et al.*, 1987; Barlozzari *et al.*, 1985; Ben-Eliyahu and Page, 1992). Few studies have reported fluctuation in NK-cell activity during the oestrous/menstrual cycle. In female mice, splenic NK activity was elevated around ovulation (Gruber *et al.*, 1988; Hrushesky *et al.*, 1988). In women, two studies have reported alterations in blood NK cytotoxicity during the menstrual cycle, although findings from these studies were not consistent (Sulke *et al.*, 1985; White *et al.*, 1982). None of the above-mentioned studies assessed the number of large granular lymphocyte (LGL)/NK cells within the population of splenocytes/monocytes tested for NK activity. Therefore, it is not clear whether changes in the number of LGL/NK cells

Correspondence: S Ben-Eliyahu
Received 20 February 1996; revised 2 July 1996; accepted 16 July 1996

or in the activity per cell account for the reported differences during the oestrous/menstrual cycle. To our knowledge, no consistent short-term *in vivo* or *in vitro* effects of oestradiol, progesterone or testosterone on NK activity have been reported. One study reported a lack of *in vitro* effects of these hormones on a cloned human NK cell line (Callewaert *et al.*, 1991), and another study, using oestradiol and tamoxifen in rats, indicated dose-dependent alterations in NK cytotoxicity (Baral *et al.*, 1991).

In the current study, we assessed the effects of the oestrous cycle and sex hormones on the number and activity levels of blood NK cells measured *in vitro*, and on the *in vivo* resistance to metastatic development of a mammary adenocarcinoma cell line, MADB106. This tumour line, syngeneic to the inbred F344 rats used in this study, metastasises only to the lungs following intravenous inoculation, and thus constitutes a convenient model of breast cancer metastasis. The use of this tumour simulates a partial but well-defined subprocess of the overall metastatic process (Barlozzari *et al.*, 1985; Ben-Eliyahu *et al.*, 1991; Ben-Eliyahu and Page, 1992). There are three major advantages in employing the MADB106 tumour model in the current study: (1) The time interval that is most critical for the establishment of metastasis is shorter than 24 h (Barlozzari *et al.*, 1985); therefore, the efficiency of the host anti-metastatic activity can be correlated with the similarly short oestrous cycle phases in the rat. (2) Host control of the metastatic process (number of metastases established rather than size) is markedly dependent on the activity levels of LGL/NK cells during the first 24 h after tumour injection (Barlozzari *et al.*, 1985; Ben-Eliyahu *et al.*, 1991; Ben-Eliyahu and Page, 1992). Therefore, the resistance to metastatic development also reflects *in vivo* levels of LGL/NK cell activity and suggests the biological significance of alterations in NK activity that we directly measure *in vitro*. (3) As will be reported here, the MADB106 tumour does not express oestrogen receptors and its *in vitro* proliferation rate appears not to be affected by oestradiol or progesterone. Therefore, the use of this tumour advances the study of the indirect effects of sex hormones on tumour development (mediated via alteration in immunity or other physiological mechanisms).

In order to maximise the overlap between the time of the highest levels of sex hormones and the NK-sensitive period of the MADB106 tumour, we chose to inoculate the rats with tumour cells at the beginning of the light phase, when there is a sharp rise in the levels of most sex hormones on pro-oestrus, and to assess the cumulative anti-metastatic activity of the host during the first 9 h after tumour inoculation. This assessment was conducted by measuring the percentage of tumour cells retained in the lungs 9 h after inoculation. We and others have reported high correlation between lung tumour cell retention within hours after tumour cell inoculation and the consequent number of lung metastases found 3 weeks later (Barlozzari *et al.*, 1985; Ben-Eliyahu and Page, 1992). Also, in various studies using this tumour model and assessing the effects of surgery, stress, morphine or ethanol on lung tumour cell retention and on the number of lung metastases detected 3 weeks later, very similar effects in these two indices were observed, and changes in NK activity paralleled changes in susceptibility to the MADB106 metastasis (Ben-Eliyahu *et al.*, 1991, 1993, 1996; Page *et al.*, 1993, 1994; 1995; Yirmiya *et al.*, 1991, 1992).

### Materials and methods

### *Animals, oestrous cyclicity and hormonal pattern*

Fischer 344 (F344) rats were purchased from Harlan Sprague Dawley (HSD), Indianapolis, USA, housed five per cage with free access to food and water and kept under a 12:12 h light–dark cycle. Before all experiments, rats were acclimatised to the vivarium for a minimum of 4 weeks. The female rat has a 4–5 day oestrous cycle consisting of the following phases/days: pro-oestrus (pe), oestrus (e), metoestrus (me), dioestrus day 1 (de1), and a variably occurring dioestrus day 2 (de2). Oestradiol and prolactin levels, unlike those of progesterone and other sex hormones, begin to increase gradually during dioestrus (Lapolt *et al.*, 1986; Nequin *et al.*, 1979). Levels of most sex hormones, including oestradiol, progesterone, prolactin, LH and follicle-stimulating hormone (FSH), rise sharply and peak during the light phase of pro-oestrus and return to baseline levels before the oestrous day (Lapolt *et al.*, 1986; Nequin *et al.*, 1979).

### *Ovariectomy*

Females were ovariectomised at 12 weeks of age under halothane anaesthesia through a midline abdominal incision (for procedure see D'Amour and Blood, 1954). Sutures were removed 10 days after surgery. Rats were allowed a minimum of 3 weeks for recovery.

### *MADB106 tumour cells*

MADB106 is a selected variant cell line obtained from a pulmonary metastasis of a mammary adenocarcinoma (MADB100) chemically induced in the inbred F344 rat (Barlozzari *et al.*, 1985). MADB106 cells were maintained in 5% carbon dioxide at 37°C in monolayer cultures in complete medium [RPMI-1640 medium (Gibco, Grand Island, NY, USA) supplemented with 10% heat-inactivated fetal bovine serum (FBS), 45 U penicillin G $ml^{-1}$, 0.045 mg streptomycin $ml^{-1}$, 2 mM L-glutamine, 0.1 mM non-essential amino acid and 1 mM sodium pyruvate] and separated from the flask (Falcon 3023) using 0.25% trypsin.

### *Radiolabelling of tumour cells and lung clearance assessment*

For lung clearance assessment, DNA radiolabelling of tumour cells was accomplished by adding 0.4 $\mu$Ci of [$^{125}$I]iododeoxyuridine ([$^{125}$I]IDUR) (ICN Radiomedicals, Irvine, CA, USA) per ml of complete media to the growing cell culture 1 day before harvesting the cells for injection.

For tumour cell injection, rats were lightly anaesthetised with halothane and $4 \times 10^5$ $kg^{-1}$ [$^{125}$I]IDUR-labelled MADB106 tumour cells were injected into the tail vein in approximately 0.5 ml phosphate-buffered saline (PBS). This procedure requires 1–2 min, after which rats quickly awake and behave normally. Nine hours later, rats were euthanised with halothane and their lungs removed to measure radioactive content in a gamma counter. Percentage radioactivity retained in the lungs is the ratio between radioactivity measured in the lungs and total radioactivity in the injected tumour cell suspension. Our previous studies with radiolabelled MADB106 tumour cells indicated that the level of radioactivity released from intact cells is negligible (Ben-Eliyahu and Page, 1992).

### *Selective* in vivo *depletion of LGL/NK cells*

Two days before tumour inoculation or blood sampling, 1.5 mg $kg^{-1}$ MAb 3.2.3 (Pittsburgh Cancer Institute) was injected intravenously (i.v.) under light halothane anaesthesia. *In vivo* treatment of rats with MAb 3.2.3 selectively depletes LGL/NK cells and eliminates NK- and antibody-dependent non-MHC-restricted cell cytotoxicity without affecting other immune functions; T-cell function and percentage of T cells, monocytes and polymorphonuclear (PMN) cells are unaffected (Chambers *et al.*, 1989, 1992; van den Brink *et al.*, 1990). In a previous study, using this dose of the MAb 3.2.3, we showed a complete abolition of blood and splenic LGL/NK cytotoxicity and a 100-fold increase in the lung retention and metastatic colonisation of MADB106 tumour cells (Ben-Eliyahu and Page, 1992). We have also used other monoclonal antibodies (R73, W3/25 and ED2), mouse serum and saline as controls for the administration of the depleting agent and found no differences between the effects of these controls and no injection (Ben-Eliyahu and Page, 1992).

*Whole blood NK cytotoxicity assay*

Rats were lightly anaesthetised with halothane, and blood was drawn into a heparinised syringe (20 units $ml^{-1}$ blood, preservative free) by cardiac puncture. Exactly 1 ml of blood was washed once with PBS (4 × blood volume) and twice with complete media (3 × blood volume, 15% FBS). For each wash, the mixture was centrifuged at 400 ***g*** for 10 min and the supernatant removed down to the original blood volume. For each of the six effector–target (E:T) ratios used, 100 $\mu l$ of washed blood was placed into a well of a microtitre plate and 150 $\mu l$ of $^{51}Cr$-labelled YAC-1 tumour cells in complete medium was then added on top of the blood. A concentration of $8 \times 10^5$ YAC-1 $ml^{-1}$ was used for the lowest E:T ratio (approximately 8:1, leucocytes: YAC-1, depending on leucocyte concentration per ml of blood) and sequentially divided by two for higher E:T ratios (approximately 256:1 at the highest). Plates were centrifuged at 500 ***g*** for 10 min to create a buffy coat layer of leucocytes and target cells above the red blood cells before a 4 h incubation period. Following incubation, plates were centrifuged again and aliquots of 100 $\mu l$ of the supernatant were recovered from each well for assessment of radioactivity in a gamma counter. The spontaneous and maximum release of radioactivity from tumour cells were measured separately for each of the six tumour concentrations, and percentage specific lysis was calculated for each E:T ratio using the standard formula: [(experimental release – spontaneous release)/(maximum release – spontaneous release)] × 100.

*Flow cytometry*

An aliquot of 100 $\mu l$ of blood was combined with 50 $\mu l$ of PBS (supplemented with 2% FBS and 0.1% sodium nitrite and 2 $\mu g$ of the MAb 3.2.3 conjugated with fluorescein isothiocyanate (Pittsburgh Cancer Institute). Samples were kept in the dark at room temperature and incubated for 15 min before 2 ml of FACs lysis solution (Becton Dickinson) was added. Ten minutes later, samples were centrifuged for 5 min at 500 ***g*** and the lysis solution aspirated. Cells were washed again with 2 ml of PBS (5 min centrifugation, 500 ***g***) and resuspended in 300 $\mu l$ of PBS for flow cytometry analysis using a FACScan (Becton Dickinson). The MAb 3.2.3 has been shown in the rat to recognise a surface antigen, NKR-P1, which is expressed exclusively on fresh and IL-2-activated LGL/NK cells (MAb $3.2.3^+$bright) and, to a much smaller degree, on polymorphonuclear (PMN) cells (MAb $3.2.3^+$dim) (Chambers *et al*., 1992). Our experience with this procedure has indicated that there is no overlap between the bright and the dim cells (Ben-Eliyahu *et al*., 1993). The number of white blood cells per ml of blood was assessed in each blood sample using a Coulter counter.

*Counterbalancing, timing of procedures and statistics*

The time and order of blood draw, drug administration and tumour injection were counterbalanced across groups and oestrous phases in all experiments. For each experiment, the procedure of either tumour inoculation or blood draw was completed within less than 2 h in all animals and was conducted during the first half of the light phase (unless otherwise noted). One factor ANOVA was conducted (repeated measures for NK cytotoxicity in experiment 4). Bonferroni *post hoc* tests or planned contrasts were used to identify specific differences. An alpha of 0.05 was set for all experiments.

*Experiment 1: The effects of the oestrous cycle on host resistance to metastasis and LGL/NK sensitivity of the metastatic process*

Oestrous cyclicity and phase were determined according to the method described in Everett (1989) based upon vaginal cellularity. Daily vaginal smears were performed in 6-month-old females (200 g, $n = 34$ cycling females) for 9 consecutive days during the first half of the light phase. On the tenth day, at the same time of vaginal cellularity sampling, each rat was inoculated with MADB106 tumour cells. To compare males with females, and to establish a relationship between the dose of tumour injected and the percentage of tumour cell retention, similarly handled age-matched males (350 g) were injected with either the same dose, one-half or twice the dose of tumour cells per body weight ($n = 6$, 5 and 5 respectively). Tumour cell retention was assessed in all rats at 9 h after tumour injection. In a replicate study, 18 cycling females were used as described above. Six were selectively depleted of LGL/NK cells before tumour injection to verify the dependence of lung clearance on LGL/NK cells in this paradigm.

*Experiment 2: The effects of oestradiol and progesterone on host resistance to metastasis in ovariectomised females*

Ovariectomised (OVX) females were injected subcutaneously (s.c.) with oestradiol benzoate (EB) or progesterone (P) at different time intervals before tumour inoculation, and lung tumour retention was measured. Ovariectomised females (210 g, 15 weeks old, $n = 57$) were randomly assigned to the following groups and were given five successive daily injections of drugs/vehicle (Figure 2). One group received vehicle injections throughout the 5 day period (control); two groups received drugs only on the fifth day, one group receiving P (6 mg $kg^{-1}$ in 0.25 ml of peanut oil), and the second group 16 $\mu g$ $kg^{-1}$ EB (in 0.25 ml of corn oil); three groups were also injected with an overall dose of 16 $\mu g$ $kg^{-1}$ EB that was given in two successive injections (8 $\mu g$ $kg^{-1}$ each), one group on days 1 and 2, the second group on days 4 and 5, and the third group also on days 4 and 5, but concomitantly with P (6 mg $kg^{-1}$) on day 5. On the sixth day, all rats were inoculated with radiolabelled MADB106 cells and euthanised with halothane 9 h later to assess lung tumour retention.

The doses of EB were chosen to induce high physiological levels of oestradiol for approximately 24–48 h after administration. In the intact female rat, average serum oestradiol levels increase from 15 pg $ml^{-1}$ on oestrous/metoestrous days to 55 pg $ml^{-1}$ on pro-oestrus day (Lapolt *et al*., 1986). The 15 pg $ml^{-1}$ level found on metoestrous day was elevated to 25 pg $ml^{-1}$ within 12 h after the administration of 4 $\mu g$ $kg^{-1}$ EB, or elevated to 160 pg $ml^{-1}$ after the administration of 80 pg $ml^{-1}$ EB. These elevated levels decreased and approached baseline levels within 24 and 48 h respectively in OVX rats (Matt *et al*., 1986). Based on these reports and approximated linear relationships between levels of EB injected and induced blood levels of oestradiol, we injected a total of 16 pg $kg^{-1}$ EB to OVX females, estimating the induced oestradiol levels to be approximately 40 pg $ml^{-1}$. The dose of progesterone used is the standard for the induction of proceptivity in OVX females (following sensitisation with oestradiol). EB and progesterone were dissolved in the specific oil vehicle indicated in the literature reporting their serum levels.

*Experiment 3:* In vitro *effects of oestradiol and progesterone on MADB106 tumour proliferation, and the levels of oestradiol and progesterone receptors on MADB106*

Oestradiol or progesterone at concentration of $10^{-8}$, $10^{-9}$, $10^{-10}$ and $10^{-11}$ M or vehicle were added to the growing culture of MADB106 cells, and cells were harvested and counted at 24 or 48 h after addition of these hormones. In a separate experiment, MADB106 tumour cells were tested for oestradiol and progesterone receptors using the standard clinical procedure described in Geier *et al*. (1982), and the MCF-7 tumour line was tested for progesterone receptors as a positive control.

*Experiment 4: Oestrous cycle and the number and activity level of circulating LGL/NK cells*

Flow cytometry was used to assess the number of blood LGL/NK cells (MAb 3.2.3$^{+}$bright) per ml of blood, and the whole blood NK cytotoxicity assay was used specifically to assess LGL/NK cytotoxicity per ml of blood. Three different assessments of whole blood NK cytotoxicity were conducted ($n$=70, 65 and 34; age=12, 16 and 20 weeks; bw=200–220 g). Blood was taken during the first half of either the dark phase (first study) or the light phase (second and third studies). To verify that the whole blood cytotoxicity assay specifically assesses LGL/NK cell-dependent cytotoxicity, rats selectively depleted of LGL/NK cells were also used in these experiments. Flow cytometry was conducted twice during the light phase, once using 20-week-old females ($n$=34, bw=220 g) and once using 10-week-old females ($n$=9, bw=210 g). Each assessment was conducted using cycling females that underwent daily vaginal cellularity sampling for the 9 days before blood withdrawal for either NK activity or flow cytometry.

### Results

*Experiment 1*

*The oestrous cycle and its effects on host resistance to metastasis* Significant differences in tumour cell retention across the 5 oestrous days were evident ($F_{(4,29)}$=4.76, $P$<0.01) (Figure 1). Specifically, rats in pro-oestrus and oestrus showed a higher percentage of tumour cell retention than rats in metoestrus and dioestrus ($P$<0.05, Bonferroni *post hoc* test). The three groups of males injected with an increasing tumour load showed an increasing percentage of tumour cell retention ($F_{(2,13)}$=4.3, $P$<0.05). Compared with the females, males injected with an equal number of tumour cells per body weight retained a lower percentage of tumour cells than females in pro-oestrus/oestrus [i.e. demonstrated a better lung clearance efficiency, ($F_{(5,34)}$=5.7, $P$<0.05, planned contrast], but similar levels of retention compared with females in metoestrus/dioestrus. The males injected with twice the dose of tumour cells per kg relative to females exhibited tumour cell retention midway between the low and high levels observed in females during metoestrus/dioestrus and pro-oestrus/oestrus respectively.

**Figure 1** The percentage of tumour cell retention in males following injections of three different tumour loads, and in females in proestrus (pe), oestrus (e), metoestrus (me), dioestrus 1 (de1) and dioestrus 2 (de2). Compared with females (—■—), males were injected with either an equal number of tumour cells per kg (—○—), half as many (- - -●- - - half tumour load) or twice as many (- - -○- - - double tumour load). Females in pro-oestrus/oestrus showed a significantly higher percentage of tumour cell retention than females in metoestrus/dioestrus. In males, the increasing tumour load resulted in a significant increase in tumour retention. Males injected with females' load of tumour cells per bw (males) retained a significantly lower percentage of tumour cells than females in proestrus/oestrus, but did not differ from females in metoestrus/dioestrus. In females, vertical bars represent s.e.m.; pe day is shown twice. Variance in the three male groups was similar to that observed in the five female groups (std=approximately 0.07%).

In the replicate study, similar oestrous effects were evident in normal cycling females (pro-oestrus/oestrus=0.56% radioactivity retained, s.e.m.=0.061; metoestrus/dioestrus=0.40%, s.e.m.=0.025; $t_{10}$=5.5, $P$<0.05). Oestrous phases were collapsed to pro-oestrus/oestrus *vs* metoestrus/dioestrus based on the findings from the first study and because of the small number of animals.

Oestrous cycles did not synchronise within or between cages in any of the studies conducted. More than 90% of females had regular 4–5 day cycles, the great majority of which had a 5 day oestrous cycle.

*The effects of selective depletion of LGL/NK cells on lung tumour retention* Selective depletion of LGL/NK cells resulted in approximately a 100-fold increase in tumour cell retention from 0.48% (s.e.m.=0.045) in normal rats to 38% in the six depleted animals (ranging from 24% to 54%). The relationship between oestrous phase and tumour retention in this small group of LGL/NK-depleted animals was not tested for methodological reasons. The purpose of the depletion was merely to verify the LGL/NK sensitivity of the tumour.

*Experiment 2*

*The effects of oestradiol and progesterone on host resistance to metastasis in OVX females* The results showed that, compared with vehicle-injected rats, OVX rats receiving 16 $\mu$g kg$^{-1}$ EB given in two equal injections on the last 2 days, but not in one injection on the last day before tumour injection, exhibited a significant increase in tumour cell retention ($F_{(5,51)}$=2.6, $P$<0.05, Bonferroni *post hoc* test) (Figure 2). No such increase was evident in the groups that

**Figure 2** The effects of oestradiol and progresterone on lung tumour cell retention in ovariectomised females. All rats were given five successive daily injections of drugs/vehicle and were inoculated with the tumour on the sixth day. Control rats (Cont) received vehicle injections only. In the five experimental groups, only drug injections are indicated in the figure and the day of each injection is specified by a subscript index. Each injection contained one of the following: progesterone (P), oestradiol benzoate (E), oestradiol benzoate and progesterone (EP) or a double dose of oestradiol benzoate (2E). Vertical bars represent s.e.m. *Significantly different from the control group ($P$<0.05).

received the same 2 day treatment with EB either 4 days before tumour injection or 2 days before tumour injection together with progesterone on day 5.

*Experiment 3*

In vitro *effects of oestradiol and progesterone on MADB106 tumour proliferation* Enumerating MADB106 tumour cells following 1 or 2 days of incubation indicated that the cells divided approximately every 18 h, and this rate was not affected by oestradiol or progesterone in the concentrations used.

*Oestradiol and progesterone receptors on MADB106* The MADB106 tumour was found to be negative for oestradiol receptors (undetectable levels) and expressed low levels of progesterone receptors ($19.4 \pm 2.1$ fmol $mg^{-1}$), which were significantly lower than in the MCF-7 tumour line ($51.6 \pm 4.6$ fmol $mg^{-1}$ ($t_8 = 6.3$, $P < 0.05$).

*Experiment 4*

*Oestrous cycle and LGL/NK cell number and activity levels* Females in pro-oestrus and oestrus exhibited a significantly greater number of LGL/NK cells (MAb $3.2.3^{+}$bright) per ml of blood compared with animals in metoestrus/dioestrus, in each of the two replicate assessments (Table I includes averages and statistics). Despite this difference in LGL/NK number per ml of blood, there were no significant oestrous phase-related differences in NK activity per ml of blood in any of the three replications conducted. In fact, very similar levels were observed in each replication (Figure 3 illustrates one replication), suggesting a lower NK activity per LGL/NK cell during pro-oestrus and oestrus.

The blood from animals that were selectively depleted of LGL/NK cells showed no MAb $3.2.3^{+}$ bright cells and no cytotoxic activity. The number of LGL/NK cells per ml of blood across the two oestrous phases was higher in 10-week-old females than in 20-week-old females (Table I) ($F_{(1,39)} = 5.4$, $P < 0.05$).

### Discussion

The statistically significant relationship between the oestrous cycle and lung clearance observed in this study indicates a decrease in host resistance to metastasis during pro-oestrus and oestrus. To provide a measure of the biological significance of the differences in lung tumour retention among oestrous phases, it is noteworthy that a difference of similar magnitude was observed between the two groups of males injected with the lowest and the highest tumour load (i.e. a 4-fold increase in tumour load), (see Figure 1). Thus, it might be suggested that during metoestrus/dioestrus the host can resist four times as many tumour cells with the same rate of success as during pro-oestrus/oestrus. Furthermore, although the absolute differences in tumour cell retention between the oestrous phases are not large, the relatively low number of animals ($n = 4-6$ per group) needed to demonstrate significant differences (in both replications) testifies to the robust nature of the phenomenon.

The effects of oestradiol benzoate (EB) and progesterone on lung tumour retention in the OVX females and the time course of these effects suggest that: (1) oestradiol administration can simulate the increase in tumour cell retention during pro-oestrus/oestrus observed in Experiment 1; (2) more than 24 h of exposure to oestradiol is needed before its full tumour-enhancing effect is evident, and this effect dissipates within less than 3 days following discontinuation of oestradiol treatment; and (3) progesterone, at the dose and timing administered, appears to attenuate the effects of oestradiol on tumour cell retention. This last suggestion is based upon the finding that progesterone administration to the EB-treated rats decreased the tumour-enhancing effects of EB by approximately 50% from a significant to a non-significant level.

In normal cycling rats, oestradiol levels begin to increase during dioestrus, 24–48 h before pro-oestrus (Lapolt *et al.*, 1986; Nequin *et al.*, 1979). Taken together, the findings in OVX and in normally cycling females, suggest that the gradual increase in oestradiol levels during the 2 dioestrous days, coupled with the surge of oestradiol during pro-oestrus, underlie the effects of the oestrous cycle on the resistance to tumour metastasis observed in Experiment 1. The termination of this effect may be initiated by the sharp increase in progesterone levels on pro-oestrus or may occur spontaneously within a day after the drop in oestradiol secretion. Nevertheless, the administration of EB or progesterone to OVX females is an imperfect simulation of the hormonal milieu of the normal cycling rat. Further studies using selective oestradiol antagonists in normal cycling rats are needed to provide additional support for this hypothesis.

**Figure 3** Mean percentage specific killing on the different days of the oestrous cycle ($\pm$s.e.m.). Cytotoxicity against different numbers of YAC-1 target cells was assessed per 0.1 ml of blood. No significant differences in cytotoxicity among oestrous days were found in this replicate or in the other two replicates conducted. Taken together with the significant alteration in the number of LGL/NK cells per ml of blood during the oestrous cycle (Table I), these findings indicate a suppressed LGL/NK cell activity per LGL/NK cell during the pro-oestrous/oestrous phase.

**Table I** Means ($\pm$s.e.m) of LGL/NK cells per ml of blood ($\times 10^4$) on different days of the oestrous cycle in two replicate studies

| | | *pe* | *e* | *me* | *de1* | *de2* | |
|---|---|---|---|---|---|---|---|
| Replicate 1 (20-week-old rats) | $n = 34$ | 27.7 (1.02) | 22.3 (1.70) | 16.8 (1.71) | 17.1 (1.69) | 20.0 (2.30) | $F_{(4,29)} = 2.81$ $P < 0.05$ |
| Replicate 2 (10-week-old rats) | $n = 9$ | 42.1 (3.68) | | 28.3 (2.55) | | | $F_{(1,7)} = 10.2$ $P < 0.05$ |

Several findings suggest that the *in vivo* effects of the oestrous cycle and its simulation by oestradiol administration in OVX females are not mediated via a direct effect of sex hormones on MADB106 tumour cells. Firstly, MADB106 cells were found to be negative for oestradiol receptors, and their proliferation rate *in vitro* was not affected by oestradiol or progesterone. Secondly, whereas 2 days, but not 1 day, of oestradiol treatment in OVX females were needed to induce its effects on MADB106 tumour retention, tumour cells were exposed to oestradiol for an equal duration in both cases (i.e. for 9 h beginning from tumour inoculation to sacrificing the animal). Thus, the impact of oestradiol on the animal rather than on the MADB106 tumour cells appears to determine host susceptibility to metastasis of this tumour. Therefore, it seems more likely that the effects of oestradiol or the oestrous cycle are mediated via some alteration in the physiological milieu that affects metastatic development.

To support the suggestion that LGL/NK cells mediate the effects of the oestrous cycle on MADB106 tumour metastasis observed in this study, it is important to establish the role of LGL/NK cells in controlling this metastatic process, and the ability of the lung clearance assay (measured by lung tumour retention) to reflect the effects of the oestrous cycle. In this study, the 100-fold increase in lung tumour cell retention in rats selectively depleted of LGL/NK cells verifies the high sensitivity of the lung clearance assay for LGL/NK cell activity. We and others have also used other approaches to implicate LGL/NK cells in controlling lung tumour retention and the consequent metastatic colonisation of the MADB106 tumour (e.g. adoptive transfer of purified LGL cells and stimulation of LGL/NK cells), and have determined that the NK-sensitive period of this process is shorter than 1 day (Barlozzari *et al*., 1985; Ben-Eliyahu and Page, 1992). As reviewed in the Introduction, the short-term assessment of lung clearance is highly predictive of the actual number of lung metastases that will grow weeks later and correlates well with levels of NK cell activity in experimental conditions (Barlozzari *et al*., 1985; Ben-Eliyahu and Page, 1992; Ben-Eliyahu *et al*., 1991, 1993, 1996; Page *et al*., 1993, 1994, 1995; Yirmiya *et al*., 1991, 1992). Thus, the lung clearance assay constitutes a short-term index of the metastatic process and maximises the overlap between the LGL/NK-sensitive period of the MADB106 metastatic process and the similarly short oestrous phases in the rat. Therefore, it could be suggested that the decrease in lung clearance observed during pro-oestrus/oestrus reflects a decrease in LGL/NK cell activity during this period of the oestrous cycle. Nevertheless, because other physiological mechanisms may, additionally or exclusively, mediate the effects of the oestrous cycle or oestradiol administration on tumour retention, more support and more direct evidence for the involvement of LGL/NK cells is needed.

To this end, we have directly assessed blood NK cell number and activity *in vitro*. Our findings indicated a significantly greater number of LGL/NK cells per ml of blood during pro-oestrus and oestrus in both replications, but no increase in LGL/NK cytotoxicity per ml of blood during these days in any of the three replications conducted. Taken together, these findings suggest that there is a decrease in blood NK activity per LGL/NK cell during pro-oestrus and oestrus. Although the blood seems to be an important immune compartment with respect to host anti-tumour NK activity in this tumour model (Ben-Eliyahu and Page, 1992), it is clear that other immune compartments (lung capillary beds, interstitial tissue and alveoli) could also play an important role. Interestingly, the same relationship between oestrous phases and LGL/NK cell number and activity was found in the spleen by Rager *et al*. (1994) using the standard splenic NK cytotoxicity assay and a different marker (5C6) for identifying LGL/NK cells. Thus, the findings of the present study in blood and those of Rager *et al*. (1994) in splenocytes suggest that sex hormones or other oestrous cycle-dependent hormonal factors induce a suppression of cytotoxicity per LGL/NK cell during pro-oestrus/oestrus, regardless of the specific location of these cells within the body. Therefore, and because these phases of the oestrous cycle were also characterised in experiment 1 by a higher susceptibility to the LGL/NK-sensitive MADB106 tumour, we hypothesise that decreased LGL/NK cell activity contributes to the observed oestrous effects on tumour metastasis.

The results of Experiment 3 also indicate that the number of LGL/NK cells per ml of blood is lower in older females (20 weeks old) than in younger females (10 weeks old) across oestrous phases (Table I). We previously conducted a longitudinal study and reported a similar decrease in females, but not in males. In the male F344 rat, LGL/NK number and activity continued to increase from 10 to 20 weeks of age (Page *et al*., 1995). This difference between the sexes may be attributed, among other things, to differences in chronic exposure to sex hormones and consequent differences in levels of various interleukins. Indeed, chronic exposure to oestradiol or progesterone under natural conditions (e.g. pregnancy) (Gabrilovac *et al*., 1988) or artificial conditions (e.g. some oral contraceptives, implants of oestradiol in ovariectomised rodents) (Baker *et al*., 1989; Hanna and Schneider, 1982; Seaman and Gindhart, 1979) was associated with suppressed NK activity, as were chronically elevated levels of prolactin (Hou and Zheng, 1988; Vidaller *et al*., 1992). Other studies have suggested a biphasic effect of oestrogens, with an early phase of increased NK activity and a long-term/higher dose reduction in NK activity (Screpanti *et al*., 1987). Thus, sex hormones may have both short-term (e.g. oestrous/menstrual) and long-term effects on LGL/NK number and activity.

Various mechanisms can mediate the effects of sex hormones on LGL/NK cell activity. Although NK cells do not appear to respond directly to sex hormones *in vitro* (Callewaert *et al*., 1991), changes in levels of interleukins, which are known to regulate LGL/NK cell activity, were reported to occur during the oestrous cycle and in response to sex hormones (Polan *et al*., 1994). The effects of sex hormones can also be mediated via interaction with the stress response. In mice, acute stress increased pituitary IL-1$\alpha$ content only during pro-oestrus (Nappi *et al*., 1994). Sex hormones and the oestrous cycle have been reported to affect baseline and stress levels of several stress hormones, including glucocorticoids (CORT), and such stress hormones are known to affect several immune functions, including NK activity (Ader *et al*., 1991). Specifically, during pro-oestrus, normal cycling rats manifest higher basal levels of CORT (Raps *et al*., 1971) and a higher CORT response to stress (Viau and Meaney, 1991). Administration of oestradiol was shown to elevate basal and stress-induced levels of CORT in ovariectomised rats (Viau and Meaney, 1991).

The findings of the current study may have implications for the surgical management of breast cancer. Although a topic of intense debate in the clinical literature, several studies have indicated differential rates of cancer recurrence (and different rates of long-term mortality) as a function of the menstrual phase during which mastectomy is performed (Badwe *et al*., 1991; Hrushesky *et al*., 1989; Saad *et al*., 1994; Senie *et al*., 1991; Spratt *et al*., 1993). Our study in rats and this clinical phenomenon have many characteristics in common. Specifically, in our study cancer development was found to be related to: (1) oestrous cyclicity; (2) the metastatic process; (3) high oestradiol/low progesterone levels; but (4) not related to a direct effect of sex hormones on the tumour. Similarly, the description of the clinical phenomenon provided by Badwe *et al*. (1991), Senie *et al*. (1991) and Saad *et al*. (1994) indicated: (1) an association between menstrual phase and the development of malignancies; (2) the involvement of the metastatic process, which is implicated by the reports that the clinical phenomenon occurred only in woman bearing tumours with evident metastatic potential (positive lymph nodes), and that mortality was associated with cancer recurrence; (3) an association of the high-risk period with increasing oestradiol levels and low progesterone levels; and (4) that the relationship between menstrual phase during which surgery is performed and subsequent metastasis occurred regardless of whether or not the excised tumour expressed receptors for sex hormones. These homologies between our findings and the suggested phenomenon in women undergoing surgery support the likelihood that this clinical phenomenon may indeed occur under certain conditions. Further, our findings suggest testable hypotheses as to the nature of the hormonal and immunological mechanisms underlying the clinical phenomenon and present an animal model that may be used to expand our understanding of this phenomenon.

**Acknowledgements**
We thank Professor Ina Weiner for her valuable discussions during the preparation of this article. This research was supported by the UCLA Program in Psychoneuroimmunology, the Israeli Ministry of Health and a Basic Research Grant from Tel Aviv University (SB-E), The Department of Veterans Affairs Medical Research Service and a gift from Trent and Mary Wells, Inc. (ANT). During the performance of some of these studies, GGP and SB-E were fellows of the UCLA program in Psychoneuroimmunology and SB-E of NIH-TS32 HD07228.

### References

ADER R, FELTEN DL AND COHEN N. (1991). *Psychoneuroimmunology*. Academic Press: San Diego.

BADWE RA, GREGORY WM, CHAUDARY MA, RICHARDS MA, BENTLEY AE, RUBENS RD AND FENTIMAN IS. (1991). Timing of surgery during menstrual cycle and survival of premenopausal women with operable breast cancer. *Lancet*, **337,** 1261–1264.

BAKER DA, SALVATORE W AND MILCH PO. (1989). Effect of low-dose oral contraceptives on natural killer cell activity. *Contraception*, **39,** 119–124.

BARAL E, KWOK S AND BERCZI I. (1991). The influence of estradiol and tamoxifen on the mixed lymphocyte reaction in rats. *Immunopharmacology*, **21,** 191–198.

BARLOZZARI T, REYNODS CW AND HERBERMAN RB. (1983). *In vivo* role of natural killer cells: involvement of large granular lymphocytes in the clearance of tumor cells in anti-asialo GM1-treated rats. *J. Immunol.*, **131,** 1024–1207.

BARLOZZARI T, LEONHARDT J, WILTROUT RH, HERBERMAN RB AND REYNOLDS CW. (1985). Direct evidence for the role of LGL in the inhibition of experimental tumor metastases. *J. Immunol.*, **134,** 2783–2789.

BEN-ELIYAHU S AND PAGE GG. (1992). *In vivo* assessment of natural killer cell activity in rats. *Prog. Neuroendocrinimmunol.*, **5,** 199–214.

BEN-ELIYAHU S, PAGE GG, YIRMIYA R, TAYLOR AN AND LIEBESKIND JC. (1993). Sympathetic modulation of natural killer cell activity and metastatic growth: a role for adrenal epinephrine. Paper presented at *Research Perspectives in Psychoneuroimmunology IV*, Boulder, Colorado, USA.

BEN-ELIYAHU S, YIRMIYA R, LIEBESKIND JC, TAYLOR AN AND GALE RP. (1991). Stress increases metastatic spread of a mammary tumor in rats: evidence for mediation by immune system. *Brain Behav. Immunol.*, **5,** 193–205.

BEN-ELIYAHU S, PAGE GG, YIRMIYA R AND TAYLOR AN. (1996). Acute alcohol intoxication suppresses natural killer cell activity *in vivo* and promotes tumor metastasis. *Nature Med.*, **2,** 457–460.

CALLEWAERT DM, MOUDGIL VK, RADCLIFF G AND WAITE R. (1991). Hormone specific regulation of natural killer cells by cortisol: direct inactivation of the cytotoxic function of cloned human NK cells without an effect on cellular proliferation. *Fed. Eur. Biochem. Soc.*, **285,** 108–110.

CHAMBERS WH, VUJANOVIC NL AND HISERODT JC. (1989). Monoclonal antibody to a triggering structure expressed on rat natural killer cells and adherent lymphokine-activated killer cell. *J. Exp. Med.*, **169,** 1373–1389.

CHAMBERS WH, BRUMFIELD AM, HANLEY-YANEZ K, LAKOMY R, HERBERMAN RB, MCCASLIN DC, OLSZOWY MW AND MCCOY P. (1992). Functional heterogeneity between NKR-P1$^{bright}$/lycopersicon esculentum lectin. (L.E.)$^{bright}$ and NKR-P1$^{bright}$/L.E.$^{dim}$ subpopulations of rat natural killer cells. *J. Immunol.*, **148,** 3658–3665.

D'AMOUR FE AND BLOOD FR. (1954). *Manual for Laboratory Work in Mammalian Physiology*. Chicago Press: Chicago.

DAVIDSON NE AND ABELOFF MD. (1993). Menstrual effects on surgical treatment for breast cancer. *Cancer Treatment Reviews*, **19,** 105–112.

EVERETT JW. (1989). *Neurobiology of Reproduction in the Female Rat*. Springer: Berlin.

GABRILOVAC J, ZADJELOVIC J, OSMAK M, SUCHANEK E, ZUPANOVIC Z AND BORANIC M. (1988). NK cell activity and estrogen hormones levels during normal human pregnancy. *Gynecol. Obstet. Invest.*, **25,** 165–172.

GEIER A, HAIMSOHN M AND LUNENFELD B. (1982). Evidence of the origin of the unoccupied oestrogen receptor in nuclei of a human breast-cancer cell line (MCF7). *Biochem. J.*, **202,** 687–691.

GORELIK E, WILTROUT RH, OKUMURA K, HABU S AND HERBERMAN RB. (1982). Role of NK cells in the control of metastatic spread and growth of tumor cells in mice. *Int. J. Cancer*, **30,** 107–112.

GRUBER SA, HOFFMAN RA, SOTHERN RB, LAKATUA D, CARLSON A, SIMMONS RL AND HRUSHESKY WJM. (1988). Splenocyte natural killer cell activity and metastatic potential are inversely dependent on estrous stage. *Surgery*, **104,** 398–404.

HANNA N AND SCHNEIDER M. (1982). Enhancement of tumor metastasis and suppression of natural killer cell activity by $\beta$-estradiol treatment. *J. Immunol.*, **130,** 974–980.

HANNA N. (1985). The role of natural killer cells in the control of tumor growth and metastases. *Biochim. Biophys. Acta*, **780,** 213–226.

HERBERMAN RB AND ORTALDO JR. (1981). Natural killer cells: their role in defenses against disease. *Science*, **214,** 24–30.

HOU J AND ZHENG WF. (1988). Effect of sex hormones on NK and ADCC activity of mice. *Int. J. Immunopharmacol.*, **10,** 15–22.

HRUSHESKY WJM, GRUBER SA, SOTHERN RB, HOFFMAN RA, LAKATUA D, CARLSON A, CERRA F AND SIMMONS RL. (1988). Natural killer cell activity: age, estrous- and circadian-stage dependence and inverse correlation with metastatic potential. *J. Natl Cancer Inst.*, **80,** 1232–1237.

HRUSHESKY WJM, BLUMING AZ, GRUBER SA AND SOTHERN RB. (1989). Menstrual influence on surgical cure of breast cancer. *Lancet*, **2,** 949–952.

LAPOLT PS, MATT DW, JUDD HL AND LU JKH. (1986). The relation of ovarian steroid levels in young female rats to subsequent estrous cyclicity and reproductive function during aging. *Biol. Reprod.*, **35,** 1131–1139.

LEVY S, HERBERMAN RB, LIPPMAN M AND D'ANGELO T. (1987). Correlation of stress factors with sustained depression of natural killer cell activity and predicted prognosis in patients with breast cancer. *J. Clin. Oncol.*, **5,** 348–353.

LYNCH EA, DINARELLO CA AND CANNON JG. (1994). Gender differences in IL-1 alpha, IL-1 beta, and IL-1 receptor antagonist secretion from mononuclear cells and urinary excretion. *J. Immunol.*, **153,** 300–306.

MATT DW, LAPOLT PS, JUDD HL AND LU JKH. (1986). Estrogen exposure affects the post-ovariectomy increase in both LH and FSH release in female rats. *Neuroendocrinology*, **42,** 21–27.

NAPPI RE, GUO AL, PETRAGLIA F, BONATI ME, CRISCUOLO M, FICARRA G, ZARA C AND GENAZZANI AR. (1994). Pituitary and ovarian interleukin-1 alpha content changes according to estrous cycle and acute stress exposure. *Gynecol. Endocrinol.*, **8,** 259–264.

NEQUIN LG, ALVAREZ J AND SCHWARTZ NB. (1979). Measurement of serum steroid and gonadotropin levels and uterine and ovarian variables throughout 4 day and 5 day estrous cycles in the rat. *Biol. Reprod.*, **20,** 659–670.

OLDHAM RK. (1990). Natural killer cells: history, relevance, and clinical applications. *Nat. Immunol. Cell Growth Regul.*, **9,** 297–312.

PAGE GG, BEN-ELIYAHU S, YIRMIYA R AND LIEBESKIND JC. (1993). Morphine attenuates surgery-induced enhancement of metastatic colonization in rats. *Pain*, **54,** 21–28.

PAGE GG, BEN-ELIYAHU S AND LIEBESKIND JC. (1994). The role of LGL/NK cells in surgery-induced promotion of metastasis and its attenuation by morphine. *Brain Behav. Immunol.*, **8,** 241–250.

PAGE GG, BEN-ELIYAHU S AND TAYLOR AN. (1995). The development of natural killer cell activity and resistance to tumor metastasis in the Fischer 344 rat. *J. Neuroimmunol.*, **63,** 69–77.

POLAN ML, DANIELE A AND KUO A. (1988). Gonadal steroids modulate human monocyte interleukin-1 (IL-1) activity. *Fertil. Steril.*, **49,** 964–968.

POLAN ML, LOUKIDES J, NELSON P, CARDING S, DIAMOND M, WALSH A AND BOTTOMLY K. (1989). Progesterone and estradiol modulate interleukin-1 beta messenger ribonucleic acid levels in cultured human peripheral monocytes. *J. Clin. Endocrinol. Metab.*, **69,** 1200–1206.

POLAN ML, KUO A, LOUKIDES J AND BOTTOMLY K. (1990). Cultured human luteal peripheral monocytes secrete increased levels of interleukin-1. *J. Clin. Endocrinol. Metab.*, **70,** 480–484.

POLAN ML, LOUKIDES JA AND HONIG J. (1994). Interleukin-1 in human ovarian cells and in peripheral blood monocytes increases during the luteal phase: evidence for a midcycle surge in the human. *Am. J. Obstet. Gynecol.*, **170,** 1000–1007.

RAPS D, BARTHE PL AND DESAULLES PA. (1971). Plasma and adrenal corticosterone levels during the different phases of the sexual cycle in normal female rats. *Experientia*, **77,** 283–291.

RATAJCZAK HV, SOTHERN RB AND HRUSHESKY WJM. (1988). Estrous influence on surgical cure of a mouse breast cancer. *J. Exp. Med.*, **168,** 73–82.

RAGER DR, COLEMAN KJ AND KLEIN SL. (1994). Cellular and humoral responses to brief acute restraint stress in rats: sex and estrous phase differences. Paper presented at the *27th Annual meeting of the Winter Conference on Brain Research.* Workshop organized by BEN-ELIYAHU S. These findings were confirmed by personal communication with Dr Rager. The marker used (5C6) is described in Jaso-Friedmann L, Leary JH, St. John AL, Harris DT, Koren HS and Evans DL. (1992). *Cell. Immunol.*, **141,** 131.

SAAD Z, BRAMWELL V, DUFF J, GIORTTI M, JORY T, HEATHCOTE G, TURNBULL I, GARCIA I AND STITT L. (1994). Timing of surgery in relation to the menstrual cycle in premenopausal women with operable breast cancer. *Br. J. Surg.*, **81,** 217–220.

SCHANTZ SP, BROWN BW, LIRA E, TAYLOR DL AND BEDDING-FIELD N. (1987). Evidence for the role of natural immunity in the control of metastatic spread of head and neck cancer. *Cancer Immunol. Immunother.*, **25,** 141–148.

SCREPANTI I, SANTONI A, GULINO A, HERBERMAN RB AND FRATI L. (1987). Estrogen and antiestrogen modulation of the levels of mouse natural killer activity and large granular lymphocytes. *Cell Immunol.*, **106,** 191–202.

SEAMAN WE AND GINDHARG TD. (1979). Effect of estrogen on natural killer cells. *Arthritis Rheumatism*, **22,** 1234–1240.

SENIE RT, ROSEN PP, RHODES P AND LESSER ML. (1991). Timing of breast cancer excision during the menstrual cycle influences duration of disease-free survival. *Ann. Intern. Med.*, **115,** 337–342.

SIMON C, PIQUETTE GN, FRANCES A, WESTPHAL LM, HEINRICHS WL AND POLAN ML. (1993). Interleukin-1 type I receptor messenger ribonucleic acid expression in human endomatrium throughout the menstrual cycle. *Fertil. Steril.*, **59,** 791–796.

SPRATT JS, ZIRNHELD J AND YANCEY JM. (1993). Breast cancer detection demonstration project data can determine whether the prognosis of breast cancer is affected by the time of surgery during the menstrual cycle. *J. Surg. Onco.*, **53,** 4–9.

SULKE AN, JONES DB AND WOOD PJ. (1985). Variation in natural killer activity in peripheral blood during the menstrual cycle. *Br. Med. J.*, **290,** 884–886.

VAN DEN BRINK MRM, HUNT LE AND HISERODT JC. (1990). *In vivo* treatment with monoclonal antibody 3.2.3 selectively eliminates natural killer cells in rats. *J. Exp. Med.*, **171,** 197–210.

VERONESI U, LUINI A, MARIANI L, DEL VECCHIO M, ALVEZ D, ANDREOLI C, GIACOBONE A, MERSON M, PACETTI G, RASELLI R AND SACCOZZI R. (1994). Effect of menstrual phase on surgical treatment of breast cancer. *Lancet*, **343,** 1545–1547.

VIAU V AND MEANEY MJ. (1991). Variations in the hypothalamic-pituitary-adrenal response to stress during the estrous cycle in the rat. *Endocrinology*, **129,** 2503–2511.

VIDALLER A, GUADARRAMA F, LLORENTE L, MENDEZ JP, LARREA F, VILLA AR AND ALARCON-SEGOVIA D. (1992). Hyperprolactinemia inhibits natural killer (NK) cell function *in vivo* and its bromocriptine treatment not only corrects it but makes it more efficient. *J. Clin. Immunol.*, **12,** 210–215.

WHITE D, JONES DB, COOKE T AND KIRKHAM N. (1982). Natural killer (NK) activity in peripheral blood lymphocytes of patients with benign and malignant breast cancer disease. *Br. J. Cancer*, **46,** 611–616.

WILTROUT RH, HERBERMAN RB, ZHANG S, CHIRIGOS MA, ORTALDO JR, GREEN KM AND TALMADGE JE. (1985). Role of organ-associated NK cells in decreased formation of experimental metastases in lung and liver. *J. Immunol.*, **134,** 4267–4274.

YIRMIYA R, SHAVIT Y, BEN-ELIYAHU S, GALE RP, LIEBESKIND JC, TAYLOR AN AND WEINER H. (1991). Modulation of immunity and neoplasia by neuropeptides released by stressors. In *Neuropeptides and Systemic Diseases.* McCubbin JA, Kaufmann PG and Nemeroff CB. (eds). pp. 261–286. Academic Press: San Diego.

YIRMIYA R, BEN-ELIYAHU S, GALE RP, SHAVIT Y, LIEBESKIND JC AND TAYLOR AN. (1992). Ethanol increases tumor progression in rats: possible involvement of natural killer cells. *Brain Behav. Immunol.*, **6,** 74–86.